# Role of tissue factor in delayed bone repair induced by diabetic state in mice

**DOI:** 10.1371/journal.pone.0260754

**Published:** 2021-12-02

**Authors:** Hiroki Ehara, Kohei Tatsumi, Yoshimasa Takafuji, Naoyuki Kawao, Masayoshi Ishida, Kiyotaka Okada, Nigel Mackman, Hiroshi Kaji

**Affiliations:** 1 Department of Physiology and Regenerative Medicine, Kindai University Faculty of Medicine, Osakasayama, Osaka, Japan; 2 Advanced Medical Science of Thrombosis and Hemostasis, Nara Medical University, Kashihara, Nara, Japan; 3 Department of Medicine, Division of Hematology, UNC Blood Research Institute, University of North Carolina, Chapel Hill, NC, United States of America; Università degli Studi della Campania, ITALY

## Abstract

**Background:**

Tissue factor (TF) is the primary activator of the extrinsic coagulation protease cascade. Although TF plays roles in various pathological states, such as thrombosis, inflammatory diseases, cancer, and atherosclerosis, its involvement in bone metabolism remains unknown.

**Materials and methods:**

The present study examined the roles of TF in delayed bone repair induced by a diabetic state in mice using wild-type (WT) and low TF-expressing (LTF) male mice. A diabetic state was induced by intraperitoneal injections of streptozotocin (STZ).

**Results:**

A prolonged diabetic state significantly reduced total and trabecular bone mineral densities (BMD) as well as cortical bone thickness in WT and LTF mice; these BMD parameters were similar between WT and LTF mice treated with or without STZ. The diabetic state induced in WT mice delayed the repair of the femur following injury. The diabetic state induced in LTF mice was associated with further delays in bone repair. In *in vitro* experiments, TF significantly decreased receptor activator of nuclear factor-κB ligand-induced osteoclast formation and osteoclastogenic gene expression in RAW264.7 cells. However, it did not affect the gene expression levels of runt-related transcription factor 2 and osterix as well as alkaline phosphatase activity in mouse primary osteoblasts.

**Conclusion:**

Low TF state was associated with enhanced bone repair delay induced by diabetic state in mice. The TF-induced suppression of bone remodeling may be a contributing factor to the protective effects of TF against delayed bone repair in a diabetic state.

## Introduction

A relationship has been reported between bone metabolism and the blood coagulation/fibrinolysis system [1–4]. We and others showed that a plasminogen deficiency suppressed bone repair in mice [2, 5]. Plasminogen activator inhibitor-1 (PAI-1) plays roles in various diseases, such as osteoporosis, diabetes, obesity, cardiovascular diseases, cancer, hepatic disease, and inflammatory disease [1]. These findings suggest the involvement of the coagulation/fibrinolysis system in bone metabolism and repair.

Tissue factor (TF, coagulation factor III) is a transmembrane protein that binds to coagulation factor VII (FVII) and converts it to activated FVII, which primarily triggers the extrinsic coagulation protease cascade [6]. Recent studies indicated that in addition to its role in the activation of the coagulation cascade, TF mediates the regulation of cellular signaling pathways and gene expression [7]. TF is expressed in various cells, particularly monocytes/macrophages and endothelial cells in an inflammatory state, and is physiologically essential for the regulation of hemostasis. It has also been implicated in pathological states, including thrombosis, inflammatory diseases, cancer, and atherosclerosis [6]. We recently reported that a TF deficiency increased alveolar hemorrhage in mice infected with influenza [8]. TF is also crucial for wound healing through its initiation of hemostatic clot formation [6]. Regarding the pathological roles of TF in bone, the involvement of TF in osteosarcoma has been demonstrated [9–12]. Daubie et al. showed that the binding of FVIIa to TF induced an increase in intracellular calcium concentrations in human osteosarcoma SaOS-2 cells [9]. TGF-β up-regulated the expression of TF in human osteosarcoma MG63 cells [11], and osteosarcoma-derived TF induced a hypercoagulation state, which enhanced the proliferation and metastasis of osteosarcoma [10]. TF levels have been associated with survival and tumor progression in patients with osteosarcoma [12]. However, the physiological roles of TF in bone metabolism and repair after fractures have yet to be clarified.

Increases have recently been reported in the number of patients with diabetes, which is associated with various complications, including nephropathy, retinopathy, neuropathy, and cardiovascular diseases. Patients with diabetes are also at an increased risk of fracture [3], and fracture healing and bone repair after bone injury are delayed in the diabetic state [13]. The mechanisms underlying delayed bone repair in the diabetic state include decreases in the mobilization of stem cells, angiogenesis, chondrogenesis, and osteogenesis [13, 14]. We previously revealed the involvement of PAI-1 in delayed bone repair induced by a diabetic state in mice [15–17]. TF antigen levels and the number of TF-positive microvesicles were found to be elevated in the blood of diabetic patients [18, 19]. However, it is important to note that several methods may be used to measure plasma TF levels, including antigen- and activity-based assays [20]. The expression of TF has also been associated with tissue repair and inflammation [6]. Collectively, these findings suggest a role for TF in the bone repair process.

There is currently no information on a complete TF deficiency in humans [21]. Homozygous TF-deleted mice die during embryonic development, which prevents the functional study of TF deficiencies in mice [22–24]. Therefore, low TF-expressing (LTF) mice have been generated by rescuing TF-deleted mice via the expression of the human TF minigene with low TF activity [25]. In the present study, we examined the roles of TF in delayed bone repair induced by a diabetic state in mice using wild-type (WT) and LTF male mice.

## Materials and methods

### Ethics statement

All mouse experiments were performed according to the Guide for the Care and Use of Laboratory Animals from the National Institutes of Health and the institutional guidelines for the use and care of laboratory animals at Kindai University. The protocol was approved by the Experimental Animal Welfare Committee of Kindai University (permit numbers: KAME-31-051). All surgeries and quantitative computed tomography (qCT) were performed under 2% isoflurane. All efforts were made to minimize suffering.

### Animals

We used male LTF and control WT mice in experiments. The generation of LTF mice was described in previous studies [25, 26]. Briefly, transgenic mice homozygous for the human TF minigene were crossed with mice heterozygous for the murine TF gene to create offspring heterozygous for both murine and human TF. In further crosses of the offspring, human TF transgenic mice homologous for the murine TF gene (WT) and with a murine TF gene deficiency (LTF) were generated. TF activity in LTF mice was approximately 1–2% of that in WT mice. Mice between 8 and 12 weeks of age were used in experiments. All mice were sacrificed by cervical dislocation under inhalation anesthesia with isoflurane after experiments.

### Diabetic mouse model

Diabetes was induced in male WT and LTF mice by daily intraperitoneal injections of streptozotocin (STZ; Sigma, St Louis, MO, USA, # S0130), a pancreatic β-cell cytotoxin, at a dose of 50 mg/kg body weight for 4 days, as previously described [2, 17]. Control mice were injected with the same volume of PBS. Four days after the last injection, blood glucose levels were measured with a glucometer (Glutest Ace, Sanwa Kagaku Kenkyusyo, Nagoya, Japan) using blood obtained by tail clipping. Mice with blood glucose levels higher than 300 mg/dL were considered to be diabetic. Two weeks after confirming the induction of diabetes, bone defect surgery was performed on the right femur of mice. Animals were maintained in standard cages on a 12-h light/dark cycle and received food and water *ad libitum*.

### Measurement of plasma TF levels

Murine TF antigen levels in the plasma of diabetic or non-diabetic mice were quantified by a Mouse Tissue Factor SimpleStep ELISA Kit (Abcam, Cambridge, UK).

### Bone injury model

Bone injury was induced in mice according to a previously described method [17]. Briefly, under inhalation anesthesia with 2% isoflurane, a 5-mm longitudinal incision was made in the anterior skin over the mid-femur of the right leg. The anterior-distal surface of the femur was then exposed by blunt dissection of the quadriceps. To make cortical bone defects in the femur, through-and-through perforations (0.9 mm) that disrupted the cortex and periosteal and endosteal surfaces were generated using a round bur (Komet®, Germany) operating at 10,000 rpm under ample irrigation with saline. Incised skin was then sutured in a sterile manner and anesthesia was discontinued.

### Quantitative computed tomography (qCT)

Mice were anesthetized using 2% isoflurane, and femurs with bone defects were scanned using a Latheta LCT-200 X-ray CT system (Hitachi Aloka Medical, Tokyo, Japan), as previously described [16]. The parameters used for CT scans were as follows: tube voltage, 50 kVp; tube current, 500 μA; integration time, 3.6 ms; axial field of view, 48 mm, with an isotropic voxel size of 48 μm. Images were generated by the integration of two signal averages for each femur. The total scan time was approximately 5 min per femur. Volume-rendered 3-dimensional CT pictures were reconstructed using VGStudio MAX2.1 software (Nihon Visual Science, Tokyo, Japan). The area of bone damage in each femur was quantified with an image-processing program using Latheta software (Hitachi Aloka Medical). A threshold density of 160 mg/cm^3^ was selected to distinguish mineralized from unmineralized tissues. The density range was calibrated daily with a manufacturer-supplied phantom. The tibia of mice were used to assess bone mineral density (BMD). In evaluations of trabecular BMD, trabecular regions of interest (ROIs) extending 96 μm distal to the end of the proximal growth plate over 1.5 mm towards the diaphysis were scanned. Cortical ROIs for cortical BMD and thickness were defined as 2.0-mm segments of the tibial mid-diaphysis. ROIs for total BMD were defined as 9600-μm segments (100 slices) from the distal end of the proximal growth plate of the tibia.

### Osteoclast formation

To induce the formation of osteoclasts in mouse monocytic Raw264.7 cells (ATCC, Manassas, VA) [27], cells were seeded on 96-well plates at 500 cells per well. Cells were cultured with αMEM, 10% fetal bovine serum (FBS), and 50 ng/mL receptor activator of nuclear factor κB ligand (RANKL) (Wako Pure Chem, Osaka, Japan) for 5 days. Cells were fixed with 10% formaldehyde, and staining for tartrate-resistant acid phosphatase (TRAP) was performed with a TRAP/ALP Stain kit (Wako Pure Chem) according to the manufacturer’s protocol. TRAP-positive multinucleated cells (MNCs) were counted as osteoclasts. To induce the formation of osteoclasts in mouse bone marrow cells [27], cells were collected from the tibiae of 8- to 10-week-old male mice. Unfractionated whole bone marrow cells were seeded on 96-well plates (1×10^5^ cells/cm^2^) and cultured in α-MEM (Wako Pure Chem) supplemented with 10% FBS, 1% penicillin/streptomycin, and 50 ng/mL macrophage colony-stimulating factor (M-CSF) (Wako Pure Chem) at 37°C for 3 days without a medium change. Cells were then cultured in α-MEM supplemented with 10% FBS, 1% penicillin/streptomycin, 50 ng/mL M-CSF, and 50 ng/mL RANKL at 37°C for an additional 4–5 days. TRAP-positive MNCs were counted as osteoclasts. To assess the effects of TF and activated FVII (FVIIa) on osteoclast formation in cells, lipidated recombinant human TF (Dade Innovin; Sysmex, Kobe, Japan) and/or recombinant human FVIIa (Haematologic Technologies, Essex Junction, VT, USA) were added to the culture medium for both experiments.

### Western blotting

Raw264.7 cells or primary osteoblasts were lysed with Cell Lysis Buffer (Cell Signaling Technology) supplemented with protease inhibitors, and the concentrations of extracted proteins in the lysates were measured using a BCA assay reagent (Pierce, Rockford, IL). Equal amounts of protein aliquots were separated on a 10% Mini-PROTEAN Tris-Glycine extended Precast Protein Gel (BioRad Laboratories, Hercules, CA) at 150 V for 1 hr, and then transferred onto PVDF membranes at 30 V for 1 hr. Membranes were blocked with Tris-Buffered saline/0.05% Tween 20 (TBS-T) containing 3% skim milk at room temperature for 1 hr and washed with TBS-T. Membranes were incubated with primary antibodies against cathepsin K (1:200; Santa Cruz Biotechnology, CA, USA, sc-48353), osterix (1:1000; Abcam, ab22552), RANKL (1:300; Bioss Antibodies, MA, USA, bs-0747R), osteoprotegerin (OPG) (1:300; Bioss Antibodies, bs-0431R), and β-actin (1:5000; Cell Signaling Technology, MA, USA, #4970) at 4°C overnight and then washed with TBS-T. Membranes were incubated with an appropriate horseradish peroxidase-conjugated secondary antibody at room temperature for 1 hr and then washed with TBS-T. Immune complexes on the membranes were visualized using ECL Western Blotting Detection Reagent (GE Healthcare Japan, Tokyo) and analyzed with Amersham Imager 600 (GE Healthcare Japan).

### Primary osteoblasts and alkaline phosphatase (ALP) activity

Primary osteoblasts were isolated from the neonatal calvariae of 3- to 6-day-old WT mice (129S/vJ 50%/C57BL/6J 50% mixed) as previously described [2, 27]. In brief, the calvariae of neonatal mice were digested using 1 mg/mL collagenase and 0.25% trypsin, and the resultant cells were then cultured in α-MEM with 10% FBS and penicillin/streptomycin. Osteoblasts were used at passage 2 in further experiments. Regarding ALP activity, primary osteoblasts were cultured in 24-well plates until confluent. Cells were then placed into α-MEM with 10% FBS and penicillin/streptomycin, cultured for another 24 hours, washed twice with PBS, lysed with distilled water (200 mL per well), and sonicated. Samples were centrifuged at 15,000 rpm at 4°C for 10 minutes and the supernatants were analyzed for ALP activity using a laboratory assay ALP kit (Wako Pure Chemical). Total protein concentrations were measured using a protein assay BCA kit (Wako Pure Chemical), as previously described [2]. Absorbance was measured at 405 nm (for ALP activity) and 562 nm (for the BCA assay) by the Multiskan GO microplate spectrophotometer (Thermo Fisher Scientific). ALP activity was defined as U/total protein (mg). To assess the effects of TF and FVIIa on the differentiation of osteoblasts, recombinant human TF (Dade Innovin; Sysmex) and/or recombinant human FVIIa (Haematologic Technologies) were added to the culture medium.

### Quantitative real-time polymerase chain reaction (PCR)

Total RNA was isolated from tissues and cells using the RNeasy Mini Kit (Qiagen, Hilden, Germany). Total RNA was reverse-transcribed into cDNA using High Capacity cDNA Reverse Transcription Kits (Thermo Fisher Scientific). The incorporation of SYBR Green into double-stranded DNA was assessed by quantitative real-time PCR using an ABI StepOne Real-Time PCR System (Applied Biosystems, Carlsbad, CA, USA), as previously described [17]. The PCR primers used are listed in Table 1. The mRNA levels of the target genes were normalized to β-actin or glyceraldehyde-3-phosphate dehydrogenase (GAPDH) mRNA levels.

**Table 1:** Primers used for real-time PCR experiments.

Gene		Primer sequence
*TF*	Forward	5’-CAATGAATTCTCGATTGATGTGG-3’
	Reverse	5’-GGAGGATGATAAAGATGGTGGC-3’
*Runx2*	Forward	5’-AAATGCCTCCGCTGTTATGAA-3’
	Reverse	5’-GCTCCGGCCCACAAATCT-3’
*Osterix*	Forward	5’-AGCGACCACTTGAGCAAACAT-3’
	Reverse	5’-GCGGCTGATTGGCTTCTTCT-3’
*ALP*	Forward	5’-ATCTTTGGTCTGGCTCCCATG-3’
	Reverse	5’-TTTCCCGTTCACCGTCCAC-3’
*Osteocalcin*	Forward	5’-CCTGAGTCTGACAAAGCCTTCA-3’
	Reverse	5’-GCCGGAGTCTGTTCACTACCTT-3’
*ColI*	Forward	5’-AACCCTGCCCGCACATG-3’
	Reverse	5’-CAGACGGCTGAGTAGGGAACA-3’
*RANKL*	Forward	5’-CACAGCGCTTCTCAGGAGCT-3’
	Reverse	5’-CATCCAACCATGAGCCTTCC-3’
*OPG*	Forward	5’-AGTCCGTGAAGCAGGAGT-3’
	Reverse	5’-CCATCTGGACATTTTTTGCAAA-3’
*TRAP*	Forward	5’-GCAACATCCCCTGGTATGTG-3’
	Reverse	5’-GCAAACGGTAGTAAGGGCTG-3’
*CTSK*	Forward	5’-GTTACTCCAGTCAAGAACCAGG-3’
	Reverse	5’-TCTGCTGCACGTATTGGAAGG-3’
*GAPDH*	Forward	5’-ACGGCAAATTCAACGGCAC-3’
	Reverse	5’-CTCCACGACATACTCAGCAC-3’
*β-Actin*	Forward	5’-TACCACAGGCATTGTGATGG-3’
	Reverse	5’-TTTGATGTCACGCACGATTT-3’

Abbreviations: TF: tissue factor, Runx2: runt-related transcription factor 2, ALP: alkaline phosphatase, ColI: type I collagen, RANKL: receptor activator of NF-κB ligand, OPG: osteoprotegerin, TRAP: tartrate-resistant acid phosphatase, CTSK: cathepsin K, GAPDH: glyceraldehyde-3-phosphate dehydrogenase.

### Statistical analysis

Data are presented as means ± standard errors of mean (SEM). Comparisons between two groups were performed with an unpaired *t*-test. A one-way ANOVA or two-way ANOVA followed by the Bonferroni post hoc test was employed for parametric multiple comparisons using GraphPad Prism 5 software. Significance was defined as P <0.05.

## Results

### Effects of TF on the induction of diabetes and diabetic osteoporosis

**Fig 1:**
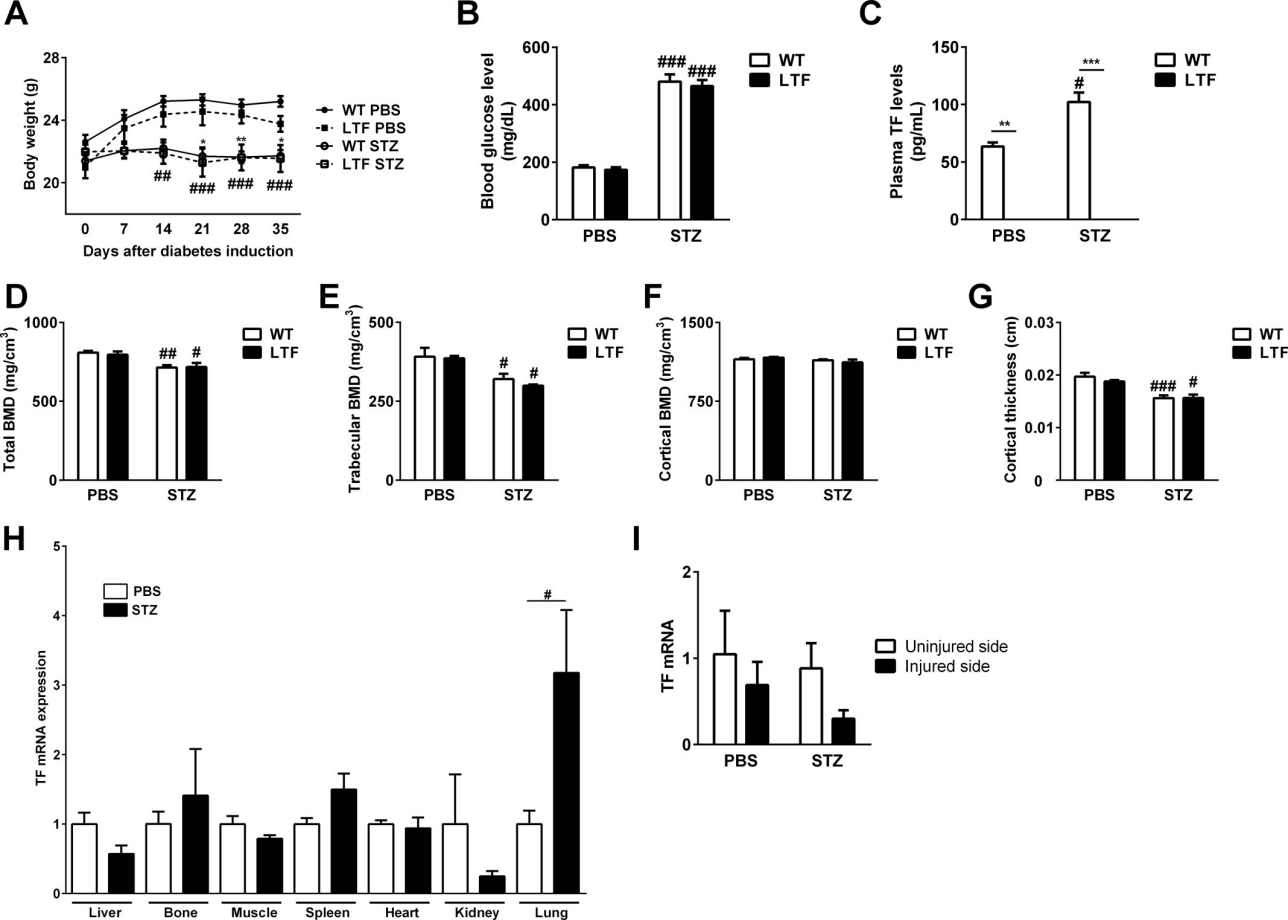
Effects of TF levels on the induction of diabetes and diabetic osteoporosis. (A) Body weight change after the induction of diabetes by a streptozotocin (STZ) injection. Data on PBS (Control: ●)- or STZ (STZ: ○)-treated WT mice and PBS (Control: ■)- or STZ (STZ: □)-treated LTF mice are shown. ^##^*P* < 0.01 and ^###^ *P* < 0.001, significantly different from PBS-treated WT control mice; *P < 0.05 and **P < 0.01, significantly different from PBS-treated LTF control mice (n≥6 in each group of mice). (B) Blood glucose levels in PBS- or STZ-treated WT and LTF mice. ^###^ *P* < 0.001, significantly different from PBS-treated WT control mice. (n≥6 in each group of mice). (C) Plasma TF levels in PBS- or STZ-treated WT and LTF mice. * *P* < 0.05. (n≥6 in each group of mice). (D-G) Total bone mineral density (BMD) (D), trabecular BMD (E), cortical BMD (F), and cortical thickness (G) in the tibia of WT and LTF mice treated with or without STZ, as assessed by qCT 3 weeks after the induction of diabetes. Data represent the mean ± SEM of 6 mice. ^#^*P* < 0.05 and ^###^*P*<0.001, significantly different from PBS-treated control mice. (H) TF mRNA levels in the liver, bone, muscle, spleen, heart, kidneys, and lungs of PBS (white bar)- or STZ (black bar)-treated WT mice. Samples were harvested 3 weeks after the induction of diabetes. Results are shown relative to β-actin mRNA values (mean ± SEM). ^#^ *P* < 0.05 (n = 4–7 in each group). (I) TF mRNA levels in the femoral bone at uninjured and injured sides in WT mice treated with or without STZ (n = 7 in each group of mice).

A diabetic state was induced in mice by intraperitoneal injections of STZ. STZ significantly decreased the body weight of both WT and LTF mice 2 or 3 weeks after the induction of the diabetic state (Fig 1A). The STZ treatment significantly increased blood glucose levels to more than 300 mg/dL in both WT and LTF mice (Fig 1B). Decreases in body weight and increases in blood glucose levels were similar in WT and LTF mice (Fig 1A and 1B). STZ significantly increased plasma TF levels in WT mice; however, TF was not detected in the plasma of LTF mice even after the induction of diabetes (Fig 1C). We then investigated the effects of the induction of diabetes on BMD. qCT analyses were performed on the tibiae of WT and LTF mice 3 weeks after the induction of diabetes. As shown in Fig 1D–1G, total BMD, trabecular BMD, and cortical thickness were significantly decreased by the induction of diabetes in both WT and LTF mice. However, no significant differences were observed between WT and LTF mice in diabetic and non-diabetic states. Therefore, TF did not affect basal BMD or diabetic osteoporosis in mice. We also examined the effects of STZ on TF expression levels in various tissues in WT mice. STZ significantly increased TF mRNA levels in lung tissues only (Fig 1H). As shown in Fig 1I, bone injury did not change TF mRNA levels at injured sites after the induction of femoral bone defects from those at the uninjured site. STZ did not affect TF mRNA levels in femoral bone, but appeared to suppress them at injured sites after the induction of femoral bone defects (Fig 1I).

### Effects of TF levels on diabetes-induced delays in bone repair

**Fig 2:**
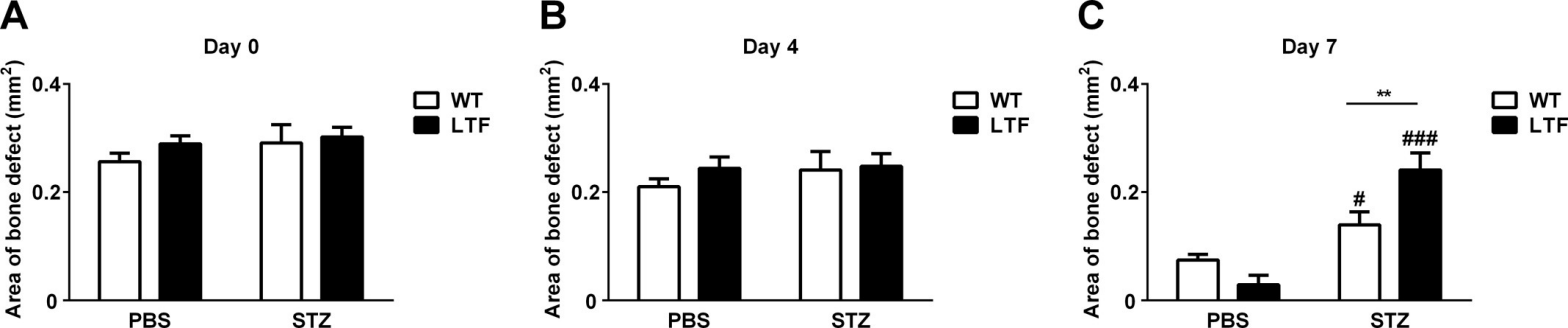
Effects of TF levels on diabetes-induced bone repair delay. (A-C) The serial quantification of bone defects at the injured site 0 (A), 4 (B), and 7 (C) days after femoral bone defects in WT (white bars) and LTF (black bars) mice treated with or without STZ, as assessed by qCT. Data represent the mean ± SEM of 6 mice. ^#^ *P* < 0.05, ^###^ *P* <0.001, significantly different from PBS-treated control mice; **P <0.01, between WT and LTF mice.

**Fig 3:**
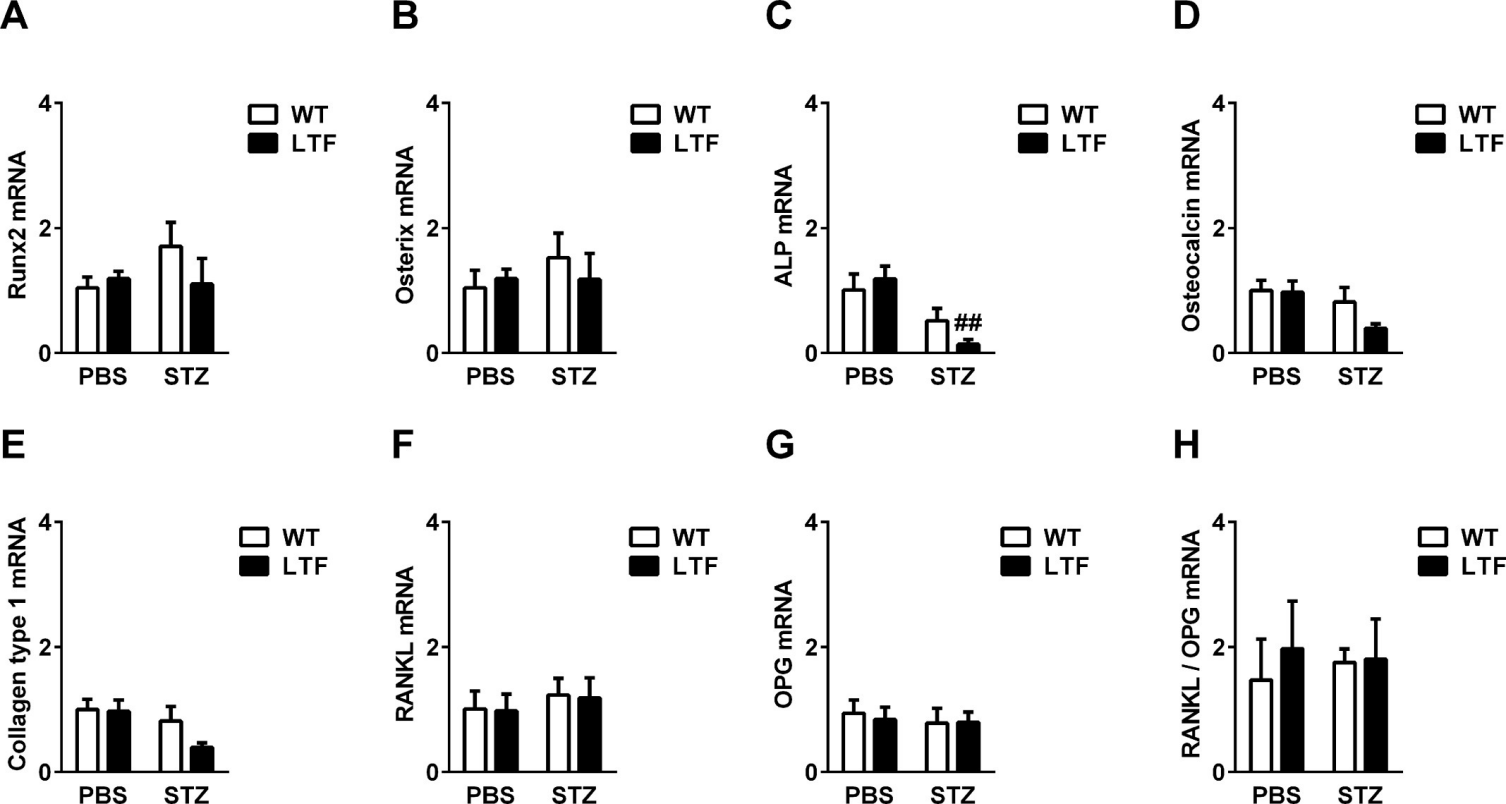
Effects of a diabetic state and TF deficiency on osteogenic and osteoclast-related gene expression. Injured femoral bone samples were extracted 7 days after bone defects from WT and LTF mice treated with or without STZ. Real-time PCR analyses of the mRNA expression levels of Runx2 (A), osterix (B), ALP (C), osteocalcin (D), type 1 collagen (E), RANKL (F), and OPG (G) were conducted, and levels were normalized to β-actin mRNA levels. The expression ratio of RANKL and OPG was also shown (H). Data represent the mean ± SEM of 6 mice. ^##^ *P* <0.01, significantly different from the PBS control.

We then investigated the bone repair process after femoral bone defects in WT and LTF mice treated with or without STZ. Bone defects were made in the lateral femoral bone of mice 2 weeks after the induction of diabetes, and bone defect areas were calculated by qCT after 0, 4, and 7 days (Fig 2A–2C). The STZ treatment induced a significant delay in bone repair 7 days after the induction of femoral bone defects in WT mice (Fig 2C). Low expression levels of TF were associated with longer delays in bone repair (Fig 2C). Injured femoral bones were extracted 7 days after the induction of bone defects and osteogenic and osteoclast-related gene expression levels were analyzed by real-time PCR (Fig 3). The mRNA levels of ALP were significantly lower in LTF mice in the diabetic state (Fig 3C), whereas those of Runx2, osterix, RANKL, and OPG and the RANKL/OPG ratio at injured sites in femoral bone in mice were not (Fig 3). The mRNA levels of osteocalcin and type 1 collagen slightly decreased in LTF mice in the diabetic state (Fig 3D and 3E).

### Effects of TF on osteoclasts

**Fig 4:**
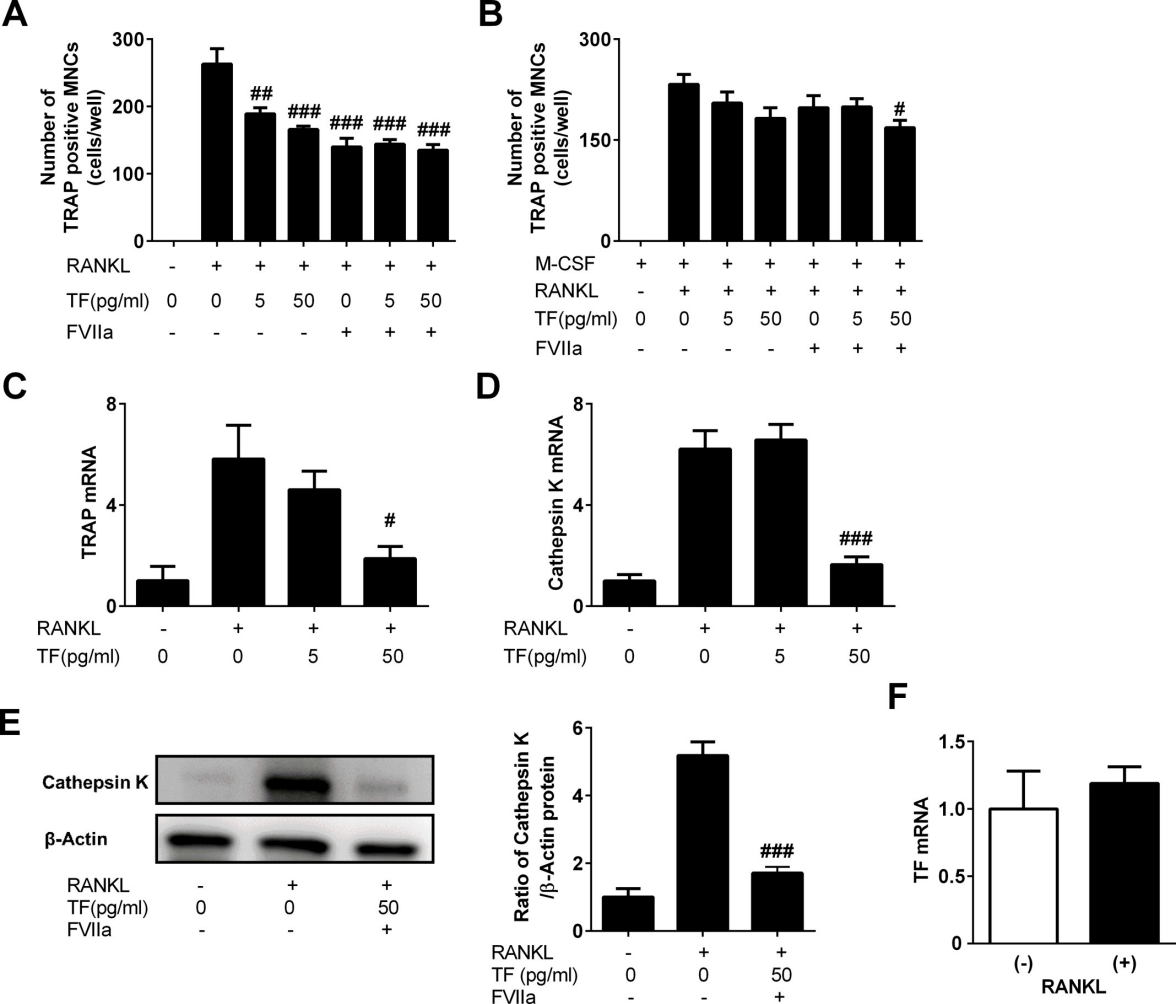
Effects of TF on osteoclast formation. (A) Osteoclast formation was induced by the treatment of Raw264.7 cells with 50 ng/mL of RANKL in the presence or absence of the indicated concentrations of TF with or without FVIIa (8 pg/mL). The number of TRAP-positive multinucleated cells (MNCs) was counted in each well. Data represent the mean ± SEM of 6 experiments in each group. ^##^ *P*<0.01, ^###^ *P*<0.001, significantly different from the RANKL only-treated control. (B) Osteoclast formation was induced by the treatment of mouse bone marrow cells with 50 ng/mL of M-CSF and 50 ng/mL RANKL in the presence or absence of the indicated concentrations of TF with or without FVIIa (8 pg/mL). The number of TRAP-positive MNCs was counted in each well. Data represent the mean ± SEM of 6 experiments in each group. ^#^ *P* <0.05, significantly different from the M-CSF and RANKL-treated control. (C-D) Real-time PCR analysis of TRAP (C) and cathepsin K (D) in 50 ng/mL of RANKL-treated RAW264.7 cells in the presence or absence of the indicated concentrations of TF. (E) Total protein was extracted from Raw264.7 cells treated with or without 50 ng/mL of RANKL in the presence or absence of TF (50 pg/mL) and FVIIa (8 pg/mL). A Western blot analysis of cathepsin K or β-actin was performed. Images represent experiments performed independently in triplicate. Protein signals were quantified by densitometry and adjusted by the density of β-actin. Data represent the mean ± SEM of 3 experiments in each group. ^###^ *P* <0.001, significantly different from the RANKL only-treated control. (F) Real-time PCR analysis of endogenous TF mRNA in Raw264.7 cells treated with or without 50 ng/mL of RANKL. Data were normalized to GAPDH mRNA expression levels, and represented as the mean ± SEM of 6 experiments in each group. ^#^ *P* <0.05, ^###^ *P* <0.001, significantly different from the RANKL only-treated control.

Since plasma TF levels were increased by the induction of diabetes and TF influenced the bone repair process in the diabetic state, we examined the effects of exogenous TF with or without FVIIa on osteoclasts *in vitro* using mouse monocytic Raw264.7 cells and mouse primary bone marrow cells. Osteoclast formation in Raw264.7 cells was induced by the addition of RANKL (50 ng/mL) for 5 days. RANKL increased the number of TRAP-positive MNCs (Fig 4A). Exogenous TF (5 and 50 pg/mL) and/or FVIIa (8 pg/mL) significantly suppressed osteoclast formation (Fig 4A). In an experiment using mouse bone marrow cells, including osteoblasts and stromal cells, RANKL increased the number of TRAP-positive MNCs, and 50 pg/mL of exogenous TF significantly decreased RANKL-induced osteoclast formation in cells in the presence of FVIIa (Fig 4B). We also quantified osteoclast-related gene expression by real-time PCR in Raw264.7 cells subjected to osteoclast formation for 5 days. RANKL increased the mRNA levels of TRAP and cathepsin K, and the addition of 50 pg/mL of exogenous TF significantly suppressed mRNA increases in both genes (Fig 4C and 4D). Moreover, TF significantly decreased RANKL-induced cathepsin K protein levels in the presence of FVIIa in Raw264.7 cells (Fig 4E). In assessments of endogenous TF, murine TF mRNA expression in Raw264.7 cells was analyzed by real-time PCR. Cells expressed TF mRNA, and its expression level was not significantly affected by RANKL-induced osteoclast formation (Fig 4F).

### Effects of TF on osteoblasts

**Fig 5:**
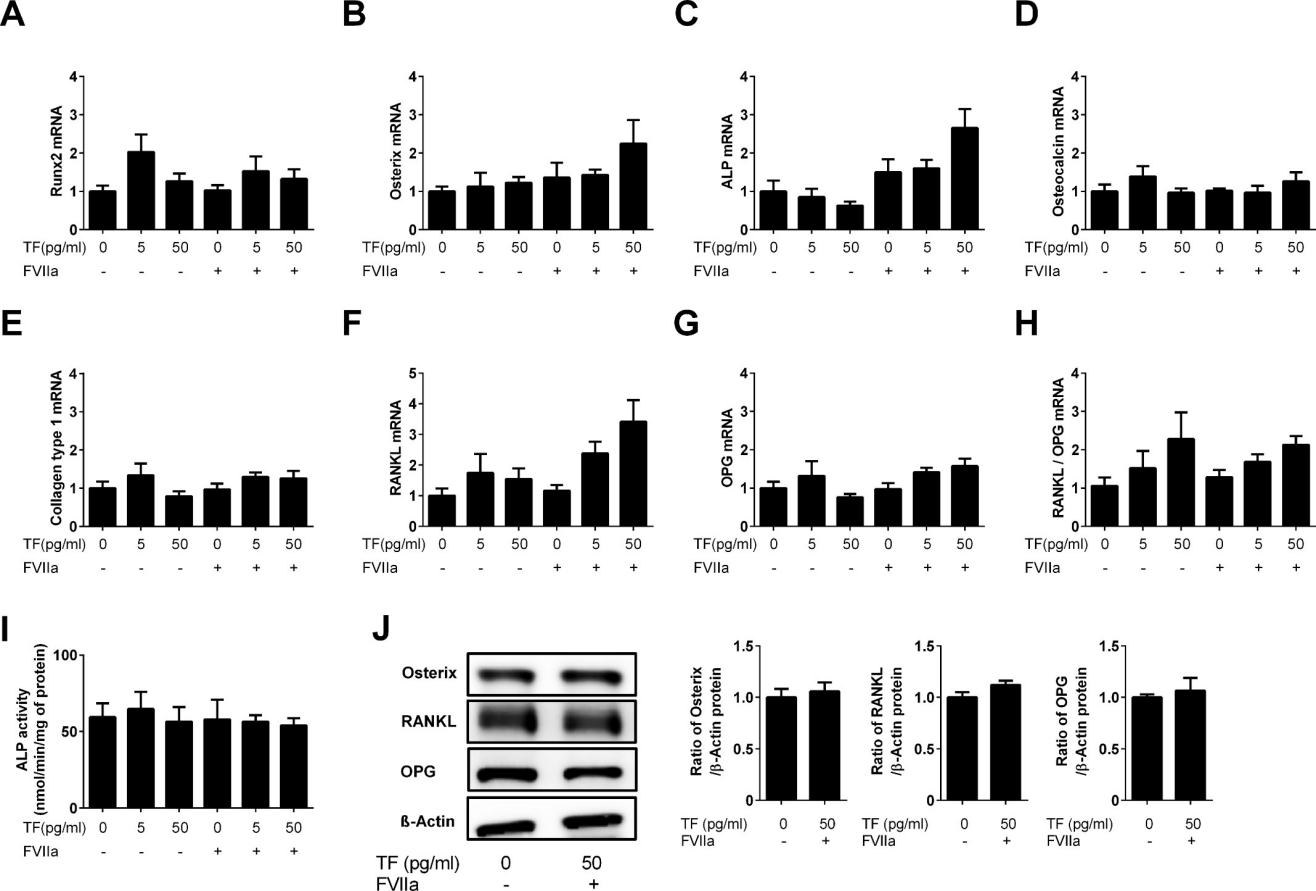
Effects of TF on the osteoblast phenotype. Mouse primary osteoblasts were isolated from the neonatal calvariae of 3- to 6-day-old WT mice, and osteogenic and osteoblast-related gene expression levels in the presence or absence of the indicated concentrations of TF with or without FVIIa (8 pg/mL) were quantified by qPCR. The mRNA levels of Runx2 (A), osterix (B), ALP (C), osteocalcin (D), type 1 collagen (E), RANKL (F), and OPG (G) and the RANKL/OPG ratio (H) were analyzed and normalized to GAPDH mRNA levels. ALP activity levels in cell homogenates were also quantified (I). Data represent the mean ± SEM of 6 experiments in each group. (J) Total protein was extracted from mouse osteoblasts treated with or without TF (50 pg/mL) and FVIIa (8 pg/mL). A Western blot analysis of osterix, RANKL, OPG, and β-actin was then performed. Images represent experiments performed independently in triplicate. Protein signals were quantified by densitometry and adjusted by the density of β-actin. Data represent the mean ± SEM of 3 experiments in each group.

Since TF influenced the bone repair process in the diabetic state, we also investigated the effects of exogenous TF (5 and 50 pg/mL) with or without FVIIa (8 pg/mL) on osteoblasts *in vitro* using mouse primary osteoblasts isolated from the neonatal calvariae of 3- to 6-day-old WT mice. Osteogenic and osteoblast-related gene expression levels in cells were quantified by real-time PCR. Neither TF nor FVIIa affected the mRNA levels of Runx2, osterix, ALP, osteocalcin, type 1 collagen, RANKL, or OPG or the RANKL/OPG ratio in primary osteoblasts (Fig 5A–5H). Consistent with the results obtained on mRNA levels, ALP activity in cell homogenates was not affected by the addition of TF and/or FVIIa (Fig 5I). Moreover, TF did not affect the protein levels of osterix, RANKL, or OPG in the presence of FVIIa in primary osteoblasts (Fig 5J).

## Discussion

The bone repair process after fractures or bone defects progresses through three phases: inflammation, restoration, and remodeling [28]. Although we previously demonstrated that modulators of the coagulation-fibrinolysis system, such as plasminogen, plasminogen activators, and PAI-1, were involved in the bone repair process after femoral bone defects in mice [2, 15–17, 29, 30], there is currently no information on the roles of TF, a principal initiator of the extrinsic coagulation cascade, in bone repair and osteoclasts. In the present study, LTF significantly enhanced diabetes-induced delays in bone repair after the induction of femoral bone defects in mice, but did not alter bone repair in a non-diabetic state. These results suggest that TF exerts some protective effects for the bone repair process suppressed by a diabetic state. Although TF is expressed in various cells, tissue inflammation enhances its expression in tissue macrophages [6]. On the other hand, diabetic and inflammatory states have been shown to increase blood TF levels despite almost undetectable blood TF levels in the physiological state [6, 18–20]. The present study showed that the diabetic state increased plasma TF levels in mice, but did not affect TF expression in bone tissues, and also that femoral bone injury did not affect the expression of TF in injured sites. These results suggest that circulating TF elevated by the diabetic state, but not local TF at bone tissues, is responsible for the protective effects of TF against delayed bone repair after bone injury induced by a diabetic state in mice.

The role of TF in bone metabolism has not yet been elucidated. The present results showed that LTF did not affect cortical or trabecular BMD in mice with or without diabetes at the uninjured site, indicating that endogenous TF is not physiologically essential for the maintenance of bone mass in mice. In an *in vitro* study, we revealed that TF significantly suppressed osteoclast formation as well as TRAP and cathepsin K expression induced by RANKL in RAW264.7 cells in the absence of FVIIa. RAW264.7 cells themselves express TF mRNA, and osteoclast formation was suppressed when only FVIIa was added. These results implicate the mechanism in which the TF-FVIIa complex affects cells through protease-activated receptors (PARs). Moreover, TF significantly suppressed RANKL-induced osteoclast formation in mouse bone marrow cells in the presence of FVIIa. The effects of TF were slightly more potent in the state with than in that without FVIIa. Therefore, TF suppressed the formation of osteoclasts partly through a FVIIa-related mechanism. The mechanisms by which TF suppresses osteoclast formation were not elucidated in the present study. Previous studies suggested that TF exerted biological effects via the interaction of FVIIa with β_1_ integrin [6, 31]. Moreover, PAR1 and PAR2 have been shown to play roles in TF-induced effects on cells [32–36]. Therefore, bone repair delays observed in LTF mice in the present study were due to a decrease in the generation of the TF-FVIIa complex and thrombin, resulting in a reduction in local signaling through PAR1 and PAR2. Further studies are needed to clarify the mechanisms by which TF suppresses osteoclast formation in mice.

Regarding the effects of TF on osteoblasts, TF did not affect ALP activity or the expression of osteogenic genes, such as osterix, ALP, and osteocalcin, in mouse primary osteoblasts. On the other hand, TF significantly suppressed the expression of ALP at the injured sites after the induction of femoral bone defects only in the diabetic state in mice, but did not affect the expression of other osteogenic genes. Although the significance of this change in the expression of ALP currently remains unknown, we cannot deny that some of the effects of TF on osteoblast phenotypes may be associated with the greater delays in bone repair induced by the diabetic state in LTF mice. Taken together with the findings showing that TF suppressed osteoclast formation *in vitro*, these results indicate that the TF-induced suppression of bone remodeling through reductions in bone resorption is involved in the protective effects of TF against delayed bone repair induced by the diabetic state in mice.

There are a number of limitations in the present study that need to be addressed. We did not show the results of a histological analysis, including ALP and TRAP staining, to assess the numbers of osteoblasts and osteoclasts due to the limited number of LTF mice available. Furthermore, we did not examine the expression of osteogenic genes at the protein level using *in vivo* samples at the damaged site. Therefore, further studies are needed to elucidate the mechanisms by which LTF contributes to further delays in bone repair induced by the diabetic state in mice.

In conclusion, the present results revealed that LTF enhanced delayed bone repair induced by a diabetic state in mice using LTF mice, but did not influence BMD. Moreover, TF suppressed osteoclast formation *in vitro*.

## References

[1] Kaji H. Adipose Tissue-Derived Plasminogen Activator Inhibitor-1 Function and Regulation. Compr Physiol. 2016;6(4):1873–96. doi: 10.1002/cphy.c160004 27783862

[2] Kawao N, Tamura Y, Okumoto K, Yano M, Matsuo O, Kaji H et al. Plasminogen plays a crucial role in bone repair. J Bone Miner Res. 2013;28(7):1561–74. doi: 10.1002/jbmr.1921 23456978

[3] Paschou SA, Dede AD, Anagnostis PG, Vryonidou A, Morganstein D, Goulis DG. Type 2 Diabetes and Osteoporosis: A Guide to Optimal Management. J Clin Endocrinol Metab. 2017;102(10):3621–34. doi: 10.1210/jc.2017-00042 28938433

[4] Rodriguez-Merchan EC, Valentino LA. Increased bone resorption in hemophilia. Blood Rev. 2019;33:6–10. doi: 10.1016/j.blre.2018.05.002 29857920

[5] Yuasa M, Mignemi NA, Nyman JS, Duvall CL, Schwartz HS, Okawa A, et al. Fibrinolysis is essential for fracture repair and prevention of heterotopic ossification. J Clin Invest. 2015;125(9):3723. 10.1172/JCI84059 PMC4588290 26325037

[6] Grover SP, Mackman N. Tissue Factor: An Essential Mediator of Hemostasis and Trigger of Thrombosis. Arterioscler Thromb Vasc Biol. 2018;38(4):709–25. doi: 10.1161/ATVBAHA.117.309846 29437578

[7] Hoffman M, Monroe DM. The multiple roles of tissue factor in wound healing. Front Biosci (Schol Ed). 2012;4:713–21. doi: 10.2741/s295 22202116

[8] Antoniak S, Tatsumi K, Hisada Y, Milner JJ, Neidich SD, Shaver CM, et al. Tissue factor deficiency increases alveolar hemorrhage and death in influenza A virus-infected mice. J Thromb Haemost. 2016;14(6):1238–48. doi: 10.1111/jth.13307 26947929 PMC5892427

[9] Daubie V, De Decker R, Nicaise C, Pochet R. Osteosarcoma cell-calcium signaling through tissue factor-factor VIIa complex and factor Xa. FEBS Lett. 2007;581(14):2611–5. doi: 10.1016/j.febslet.2007.04.078 17509570

[10] Ichikawa J, Cole HA, Magnussen RA, Mignemi NA, Butler M, Holt GE, et al. Thrombin induces osteosarcoma growth, a function inhibited by low molecular weight heparin in vitro and in vivo: procoagulant nature of osteosarcoma. Cancer. 2012;118(9):2494–506. doi: 10.1002/cncr.26518 21953059

[11] Saito M, Ichikawa J, Ando T, Schoenecker JG, Ohba T, Koyama K, et al. Platelet-Derived TGF-beta Induces Tissue Factor Expression via the Smad3 Pathway in Osteosarcoma Cells. J Bone Miner Res. 2018;33(11):2048–58. doi: 10.1002/jbmr.3537 29949655

[12] Tieken C, Verboom MC, Ruf W, Gelderblom H, Bovee JV, Reitsma PH, et al. Tissue factor associates with survival and regulates tumour progression in osteosarcoma. Thromb Haemost. 2016;115(5):1025–33. doi: 10.1160/TH15-07-0541 26763081 PMC5428546

[13] Retzepi M, Donos N. The effect of diabetes mellitus on osseous healing. Clin Oral Implants Res. 2010;21(7):673–81. doi: 10.1111/j.1600-0501.2010.01923.x 20465554

[14] Ko KI, Coimbra LS, Tian C, Alblowi J, Kayal RA, Einhorn TA, et al. Diabetes reduces mesenchymal stem cells in fracture healing through a TNFalpha-mediated mechanism. Diabetologia. 2015;58(3):633–42. doi: 10.1007/s00125-014-3470-y 25563724 PMC4346353

[15] Mao L, Kawao N, Tamura Y, Okumoto K, Yano M, Kaji H et al. Plasminogen activator inhibitor-1 is involved in impaired bone repair associated with diabetes in female mice. PLoS One. 2014;9(3):e92686. doi: 10.1371/journal.pone.0092686 24651693 PMC3961397

[16] Okada K, Okamoto T, Okumoto K, Takafuji Y, Kawao N, Kaji H et al. PAI-1 is involved in delayed bone repair induced by glucocorticoids in mice. Bone. 2020;134:115310. doi: 10.1016/j.bone.2020.115310 32142912

[17] Shimoide T, Kawao N, Tamura Y, Okada K, Okumoto K, Kaji H et al. Role of Macrophages and Plasminogen Activator Inhibitor-1 in Delayed Bone Repair in Diabetic Female Mice. Endocrinology. 2018;159(4):1875–85. doi: 10.1210/en.2018-00085 29534207

[18] Jasser-Nitsche H, Haidl H, Cvirn G, Pohl S, Gallistl S, Frohlich-Reiterer E, et al. Increased tissue factor activity promotes thrombin generation at type 1 diabetes onset in children. Pediatr Diabetes. 2020. doi: 10.1111/pedi.13086 32691481 PMC7589270

[19] Zhang C, Ou Q, Gu Y, Cheng G, Du R, Yuan L, et al. Circulating Tissue Factor-Positive Procoagulant Microparticles in Patients with Type 1 Diabetes. Diabetes Metab Syndr Obes. 2019;12:2819–28. doi: 10.2147/DMSO.S225761 32021345 PMC6978680

[20] Key NS, Mackman N. Tissue factor and its measurement in whole blood, plasma, and microparticles. Semin Thromb Hemost. 2010;36(8):865–75. doi: 10.1055/s-0030-1267040 21049387

[21] Schulman S, El-Darzi E, Florido MH, Friesen M, Merrill-Skoloff G, Brake MA, et al. A coagulation defect arising from heterozygous premature termination of tissue factor. J Clin Invest. 2020;130(10):5302–12. doi: 10.1172/JCI133780 32663190 PMC7524505

[22] Bugge TH, Xiao Q, Kombrinck KW, Flick MJ, Holmback K, Danton MJ, et al. Fatal embryonic bleeding events in mice lacking tissue factor, the cell-associated initiator of blood coagulation. Proc Natl Acad Sci U S A. 1996;93(13):6258–63. doi: 10.1073/pnas.93.13.6258 8692802 PMC39009

[23] Carmeliet P, Mackman N, Moons L, Luther T, Gressens P, Van Vlaenderen I, et al. Role of tissue factor in embryonic blood vessel development. Nature. 1996;383(6595):73–5. doi: 10.1038/383073a0 8779717

[24] Toomey JR, Kratzer KE, Lasky NM, Stanton JJ, Broze GJ, Jr. Targeted disruption of the murine tissue factor gene results in embryonic lethality. Blood. 1996;88(5):1583–7. 8781413

[25] Parry GC, Erlich JH, Carmeliet P, Luther T, Mackman N. Low levels of tissue factor are compatible with development and hemostasis in mice. J Clin Invest. 1998;101(3):560–9. doi: 10.1172/JCI814 9449688 PMC508598

[26] Pedersen B, Holscher T, Sato Y, Pawlinski R, Mackman N. A balance between tissue factor and tissue factor pathway inhibitor is required for embryonic development and hemostasis in adult mice. Blood. 2005;105(7):2777–82. doi: 10.1182/blood-2004-09-3724 15598816

[27] Takafuji Y, Tatsumi K, Ishida M, Kawao N, Okada K, Kaji H. Extracellular vesicles secreted from mouse muscle cells suppress osteoclast formation: Roles of mitochondrial energy metabolism. Bone. 2020;134:115298. doi: 10.1016/j.bone.2020.115298 32092478

[28] Claes L, Recknagel S, Ignatius A. Fracture healing under healthy and inflammatory conditions. Nat Rev Rheumatol. 2012;8(3):133–43. doi: 10.1038/nrrheum.2012.1 22293759

[29] Kawao N, Tamura Y, Horiuchi Y, Okumoto K, Yano M, Kaji H, et al. The Tissue Fibrinolytic System Contributes to the Induction of Macrophage Function and CCL3 during Bone Repair in Mice. PLoS One. 2015;10(4):e0123982. doi: 10.1371/journal.pone.0123982 25893677 PMC4404328

[30] Kawao N, Tamura Y, Okumoto K, Yano M, Matsuo O, Kaji H et al. Tissue-type plasminogen activator deficiency delays bone repair: roles of osteoblastic proliferation and vascular endothelial growth factor. Am J Physiol Endocrinol Metab. 2014;307(3):E278–88. doi: 10.1152/ajpendo.00129.2014 24918201

[31] Rothmeier AS, Liu E, Chakrabarty S, Disse J, Mueller BM, Ostergaard H, et al. Identification of the integrin-binding site on coagulation factor VIIa required for proangiogenic PAR2 signaling. Blood. 2018;131(6):674–85. doi: 10.1182/blood-2017-02-768218 29246902 PMC5805488

[32] Coughlin SR. Thrombin signalling and protease-activated receptors. Nature. 2000;407(6801):258–64. doi: 10.1038/35025229 11001069

[33] O’Neill KR, Stutz CM, Mignemi NA, Cole H, Murry MR, Nyman JS, et al. Fracture healing in protease-activated receptor-2 deficient mice. J Orthop Res. 2012;30(8):1271–6. doi: 10.1002/jor.22071 22247070

[34] Sato N, Ichikawa J, Wako M, Ohba T, Saito M, Sato H, et al. Thrombin induced by the extrinsic pathway and PAR-1 regulated inflammation at the site of fracture repair. Bone. 2016;83:23–34. doi: 10.1016/j.bone.2015.10.005 26475502

[35] Schaffner F, Ruf W. Tissue factor and PAR2 signaling in the tumor microenvironment. Arterioscler Thromb Vasc Biol. 2009;29(12):1999–2004. doi: 10.1161/ATVBAHA.108.177428 19661489 PMC2806842

[36] Smith R, Ransjo M, Tatarczuch L, Song SJ, Pagel C, Morrison JR, et al. Activation of protease-activated receptor-2 leads to inhibition of osteoclast differentiation. J Bone Miner Res. 2004;19(3):507–16. doi: 10.1359/JBMR.0301248 15040840

